# Emamectin Benzoate Treatment of Hybrid Grouper Infected With Sea Lice in Hong Kong

**DOI:** 10.3389/fvets.2021.646652

**Published:** 2021-02-12

**Authors:** Sophie St-Hilaire, Tzu Hsuan Cheng, Stephen Chi Ho Chan, Chi Fai Leung, Ka Man Chan, Kwok Zu Lim, William Furtado, Giana Bastos Gomes

**Affiliations:** ^1^Department of Infectious Diseases and Public Health, Jockey Club College of Veterinary Medicine and Life Sciences, City University of Hong Kong, Hong Kong; ^2^Temasek Life Sciences Laboratory, 1 Research Link, National University of Singapore, Singapore, Singapore

**Keywords:** grouper aquaculture, copepodid parasites, *Caligus*, parasite control, emamectin benzoate

## Abstract

Sea lice (Copepoda: Caligidae) are ectoparasites which negatively impact marine aquaculture species around the world. There are a limited number of treatments licensed for use against sea lice in tropical and semi-tropical farmed fish species. Emamectin benzoate (EB) was an effective pharmaceutical drug against sea lice infestations in several salmon industries before resistance to the product developed. This drug has not been extensively tested in marine fish within Asia. The objective of this study was to determine whether this drug could be used to treat oral infections with sea lice in hybrid grouper (*Mycteroperca tigris* × *Epinephelus lanceolatus*) cultured in saltwater net-pen sites in Hong Kong. We observed an overall reduction in sea lice infections over time, starting on the last day of the treatment up to the end of our study (i.e., 14 days after the last EB treatment). We also observed a large variation in concentrations of EB in fish on the last day of the treatment, which provides an explanation for the variation in response to the treatment. It also suggests that distribution of the medication to fish in saltwater net-pens is difficult, especially when medication is hand-mixed in the feed and possibly unevenly distributed in the daily rations. Overall, this study provides preliminary evidence that EB could be used to treat sea lice found in Hong Kong and potentially in other regions of SE Asia.

## Introduction

Sea lice are copepod parasites of economically important marine finfish found around the world. Some sea lice species affect only certain species of fish (i.e., *Lepeophtheirus salmonis*), while others, such as those belonging to the genus *Caligus*, are known to be more generalists (1, 2). *Caligus* species can live in a wide range of environments (1, 2). The larval stages of *Caligus* species can survive up to 8 days without food in temperatures ranging from 19 to 26°C (tropical and semi-tropical regions) in both marine and brackish waters (1, 2). In Asia, several species of *Caligus* sea lice, including *Caligus epidemicus, C. chiastos, C. punctatus, C. multispinosus*, and C. *rotundigenitalis* have been reported to infect farmed grouper (3–5).

**Figure 1:**
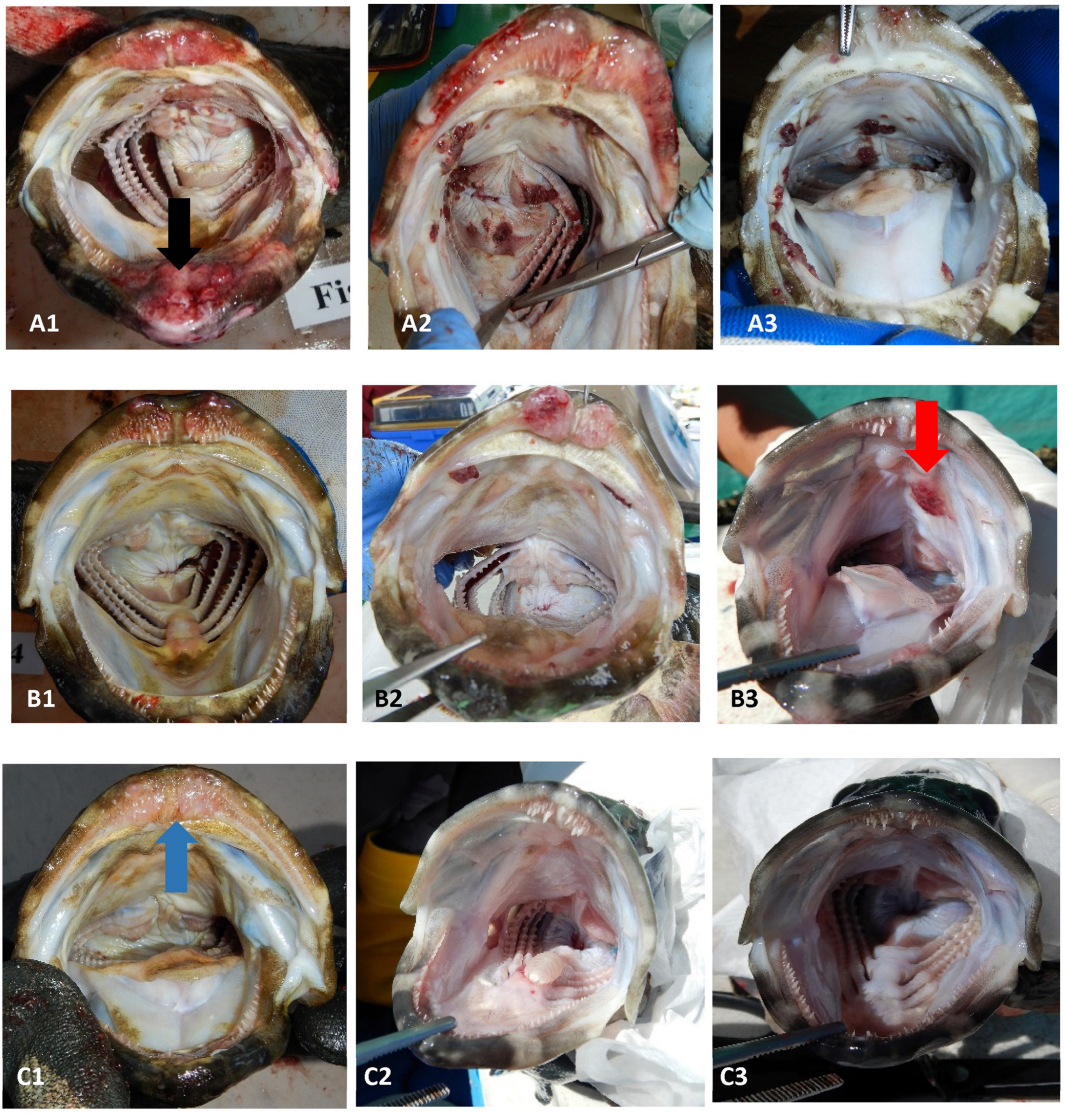
Examples of the range of sea lice infections observed on farms during the emamectin trial. Fish **(A1–A3)** were considered moderate to severe infections (category 2). Fish **(B1–B3)** were considered mild infections (category 1). Fish **(C1–C3)** were considered fish that were not infected or recovered from infection. The black arrow (on fish **A1**) illustrates a chronic lesion associated with sea lice. The red arrow (on fish **B3**) illustrates sea lice clusters (newer infections) with no tissue response. The blue arrow (on fish **C1**) illustrates tissue response in fish which was no longer infected with sea lice (post-treatment).

In Hong Kong, which has a family-based marine aquaculture industry that produces ~1,000 tons of fish per year (https://www.gov.hk/en/about/abouthk/factsheets/docs/agriculture.pdf) there have been anecdotal reports of unknown species of sea lice affecting farmed hybrid grouper

(*Mycteroperca tigris* × *Epinephelus lanceolatus*) for several years. Unlike the *Caligus* spp. that infect salmon, the sea lice found in Hong Kong are smaller (i.e., 2–4 mm as adults) and they are predominantly localized in the oral cavity of the grouper (Figure 1). Infections caused by the sea lice species found in Hong Kong can be severe, cause fish to go off-feed, and lead to mortality (A. Leung, Agriculture, Fisheries and Conservation Department Hong Kong, 2020, Personal Communication). Formalin baths have been used to control this parasite in saltwater net-pens in Hong Kong on a small scale (A. Leung, Agriculture, Fisheries and Conservation Department Hong Kong, 2020, Personal Communication). This product is a carcinogen so these treatments are difficult to administer, and like most bath treatments the effect is not long lasting. Further, because the sea lice in grouper from Hong Kong are located inside the oral cavity, bath treatments are not always as effective as in the salmon industry, where sea lice species are predominantly found on the external surface of fish.

Systemic treatments with a long residual effect, such as with emamectin benzoate (EB), and Teflubenzuron, are not widely used to treat sea lice in Hong Kong, despite their frequent use in many salmon industries around the world (6–9). Orally fed EB is distributed in different tissues including the mucus of the fish (10, 11). The drug is taken up by sea lice when it ingests mucus and blood from treated fish. The product disrupts chloride ion movement in nerve cells by blocking glutamate-gated (GluCl) and γ-aminobutyric acid (GABA)-gated (GABA-Cl) chloride channels, which eventually results in the death of the sea lice (12, 13). When EB was first introduced on the market several decades ago the product was very effective against sea lice (*Caligus* spp. and *L. salmonis*) infecting salmon; however, in the last 10 years there have been reports of resistance to EB in most salmon industries around the world (14, 15). The objective of this study was to assess the efficacy of EB on local sea lice parasites infecting the oral cavity of hybrid grouper in Hong Kong saltwater net-pen sites. We report results of sea lice treatments on five independently managed farms in bays surrounding Hong Kong.

## Materials and Methods

### Farm Selection

Hybrid grouper farms around several fish farming regions in Hong Kong affected by sea lice were identified through the diagnostic services of the City University of Hong Kong aquatic animal veterinary service. Farmers were asked to participate in an EB field trial. Farmers participating in our study had between 3 and 10 small (3m by 3m by 3m) net-pens of hybrid grouper. The criteria for inclusion in this study were: (1) that fish had sea lice infections (i.e., microscopic evidence of copepodid parasites in the oral cavity) and no bacteria or other parasitic pathogens as determined by our initial diagnostic work-up; (2) fish were not to be harvested for 21 days post treatment; (3) farms had no crustacean aquaculture species on the site with the hybrid grouper; (4) fish were still eating; and (5) the farmer was willing to withhold at least 30 untreated fish in a separate pen as controls for comparison up to 21 days from the start of the treatment. Our initial diagnostic work-up consisted of a gross necropsy, wet mount scraping of the oral cavity lesions and the gills, and a bacterial culture from the kidney of the fish on blood agar, marine agar (2% salt), and tryptic soy Agar. Gills were examined for evidence of other parasites (i.e., ciliated protozoans, monogeneans, etc) and the kidney bacterial culture was to verify that fish did not have co-infections with bacterial pathogens.

### Field Trial Emamectin Benzoate (EB)

**Figure 2:**
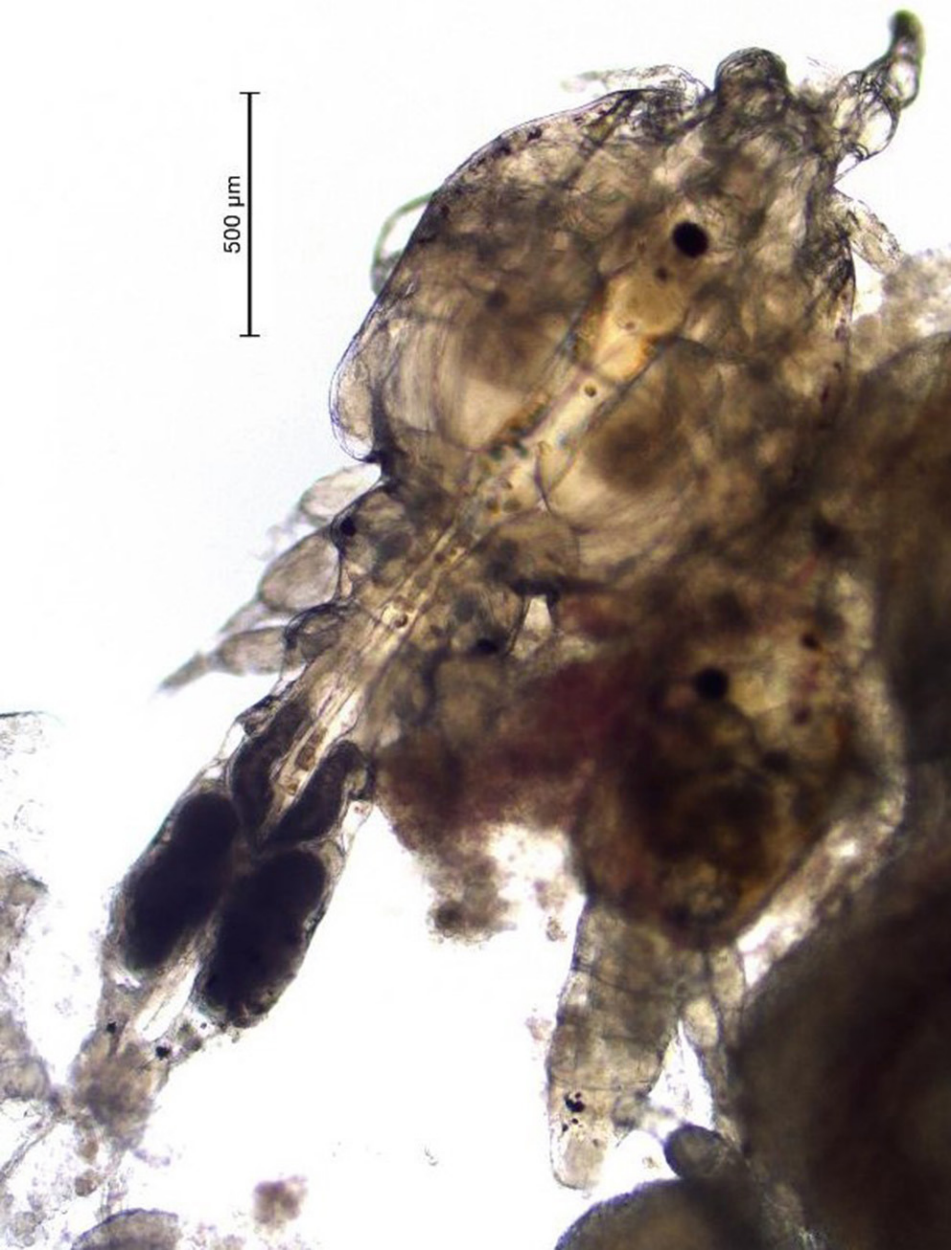
Photo of sea lice found on hybrid grouper during our study period.

Once a farmer agreed to participate in our trial and met the inclusion criteria, five fish were collected, using a dip net (outside their regular feeding period) prior to the start of the administration of EB for a baseline sample (day 0 of experiment). Fish were euthanized with an Ikigun (Auckland, New Zealand), weighed, and externally examined for the presence of sea lice. We used a 3-point photographic scale to qualitatively categorize the severity of sea lice infestation (Figure 1) because the sea lice infecting grouper were too small to grossly quantify numerically (adults were between 2 and 4 mm) (Figure 2). The tissue reaction associated with the sea lice infections also obscured the parasites making it difficult to quantify (Figure 1). A fish was considered to have a category 1 level infection if there were three or fewer small (<1 cm) lesions with parasites in its oral cavity. A fish was considered to have a category 2 infection if there were more than three small lesions, or if the lesions in its oral cavity were larger than 1 cm in diameter.

Once a farm was confirmed to only have sea lice infections and no comorbidities (i.e., other parasites and or bacterial infections), we prescribed SLICE® (Merck & Co., Inc., Kenilworth, NJ, USA.) (0.2% EB) at the dose recommended for salmon: 50 μg EB/kg for 7 days. We monitored one pen of treated fish and one pen of untreated fish on each farm included in our study. The control fish (untreated) were usually from the same pen and were maintained in a small enclosure close to the treated animals. The number of fish in the treated pens across five farms ranged between 750 and 1,350, and the weight of the fish included in our study ranged from 0.2 to 2 kg depending on the farm. The untreated pens contained between 30 and 100 fish. The treated fish on each farm were only compared to the control fish on the same farm to control for exposure level. After 21 days post treatment, we offered to treat all control animals with EB.

We calculated the inclusion rate of the medication in the feed based on a 1% feeding rate, the number of fish being treated, and their weight. The EB medication (SLICE®) was hand-mixed in the existing commercial pelleted feed used by the farmer and top coated with canola oil. The veterinarians involved in the study mixed the 1st day of medication with the farmer to demonstrate the procedure. Farmers were asked to keep records of fish mortality in the treated and control fish groups. Water temperature and salinity were recorded on the days of fish sampling.

We randomly sampled five treated fish and five untreated fish on the 7th day of the treatment, and on days 14 and 21. All fish were collected using a dip net (outside of their regular feeding time). We used feed pellets to attract the fish population to the surface to collect our sample. We photographed the oral cavity of all fish and ranked their infection based on a 3-point scale (Figure 1). We calculated the proportion of treated and untreated fish with light infections (category 1) and with moderate to severe (category 2) infections at each sampling time point. Due to limited amount of fish with moderate infections (i.e., there were only 49 category two fish out of 189 fish sampled from five farms), we dichotomized the infection data (combined category 1 and 2) to statistically compare whether the proportions of fish with any infections differed between treated and untreated groups at different time points separately. We assessed whether these proportions differed significantly using a mixed effect logistic regression model with treatment as the fixed effect, and farm as the random effect. All analyses were conducted in STATA 15.0 (Stata Corp LLC, College Station TX USA).

### Emamectin Benzoate (EB) Concentration Test

Muscle and skin tissues were collected from the same fish sampled for sea lice assessment on days 0, 7, and 21, on four out of the five farms^1^ for EB analysis using liquid chromatography with tandem mass spectrometry (LC-MS-MS) as described in Lehotay (16). The samples were submitted frozen at −80^0^C to a commercial laboratory (Chemical Testing Services, Faculty of Science, Hong Kong Baptist University, Hong Kong) for analysis. On the days when we sampled for LC-MS-MS we euthanized the fish with an Ikigun (Auckland, New Zealand). Tissue samples were frozen at −20°C and tested for EB at the end of the study. On the two last farms included in this study, we also sampled five treated fish on day 28 after the start of the treatment for EB tissue concentration in case the residue period was longer than expected. We graphically illustrate the EB tissue concentration in treated fish over time.

## Results

**Figure 3:**
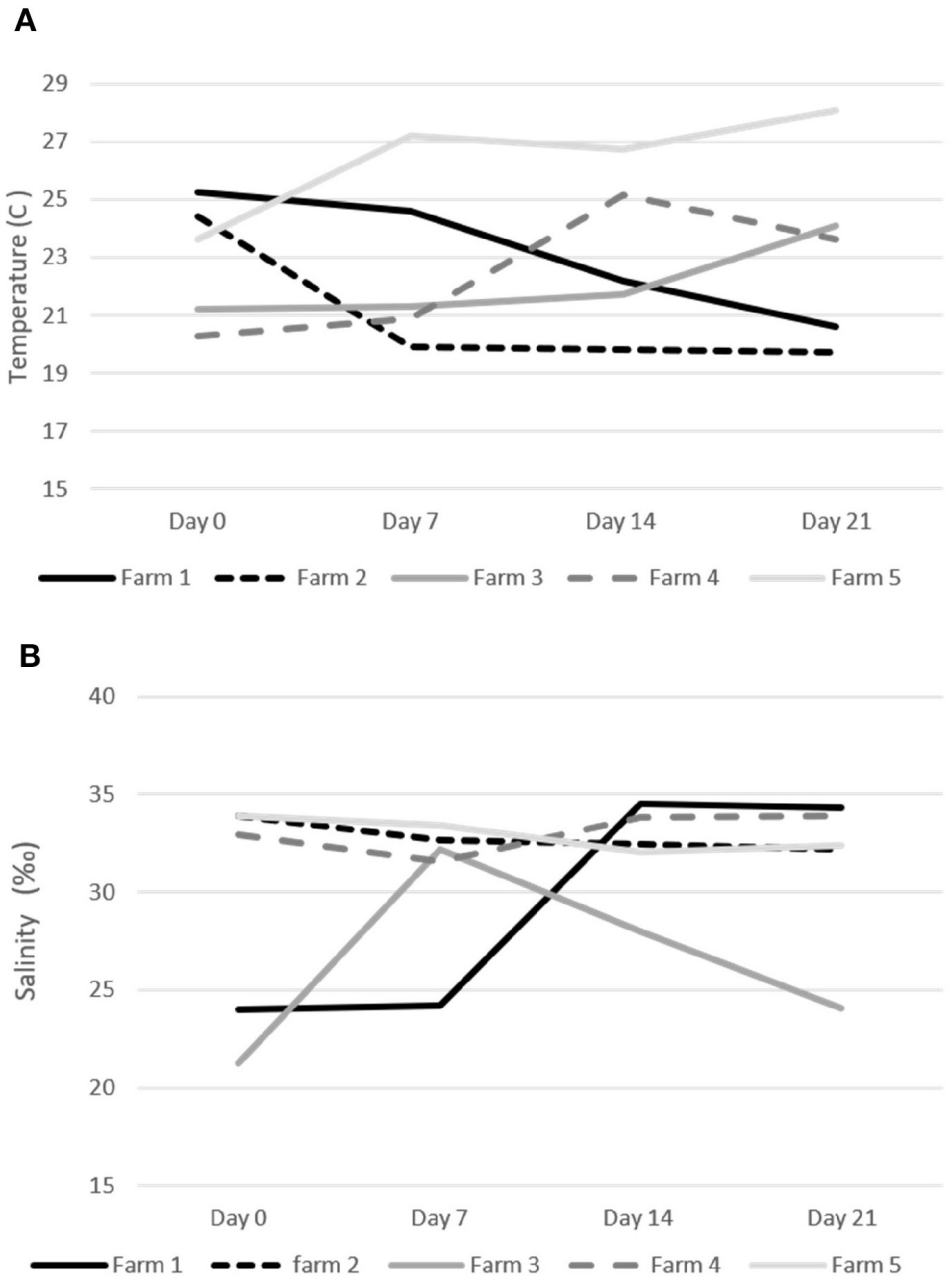
Water temperature (C) and salinity (‰) during the emamectin benzoate sea lice trails. **(A)** Water temperature on specific farms. **(B)** Water salinity on specific farms.

Water temperature during the treatments on the five study sites ranged from a minimum of 19.7°C to a maximum of 29.1°C (Figure 3). Salinity was always above 30%0 on three farms (#2, 4, and 5) and fluctuated between 21 and 32%0 on the other two sites (#1 and 3) (Figure 3). Only fish farms with confirmed sea lice infections in the oral cavity and no other pathogens on our initial health check were included in our trial. Farmers did not report any fish mortality during the trial period.

**Figure 4:**
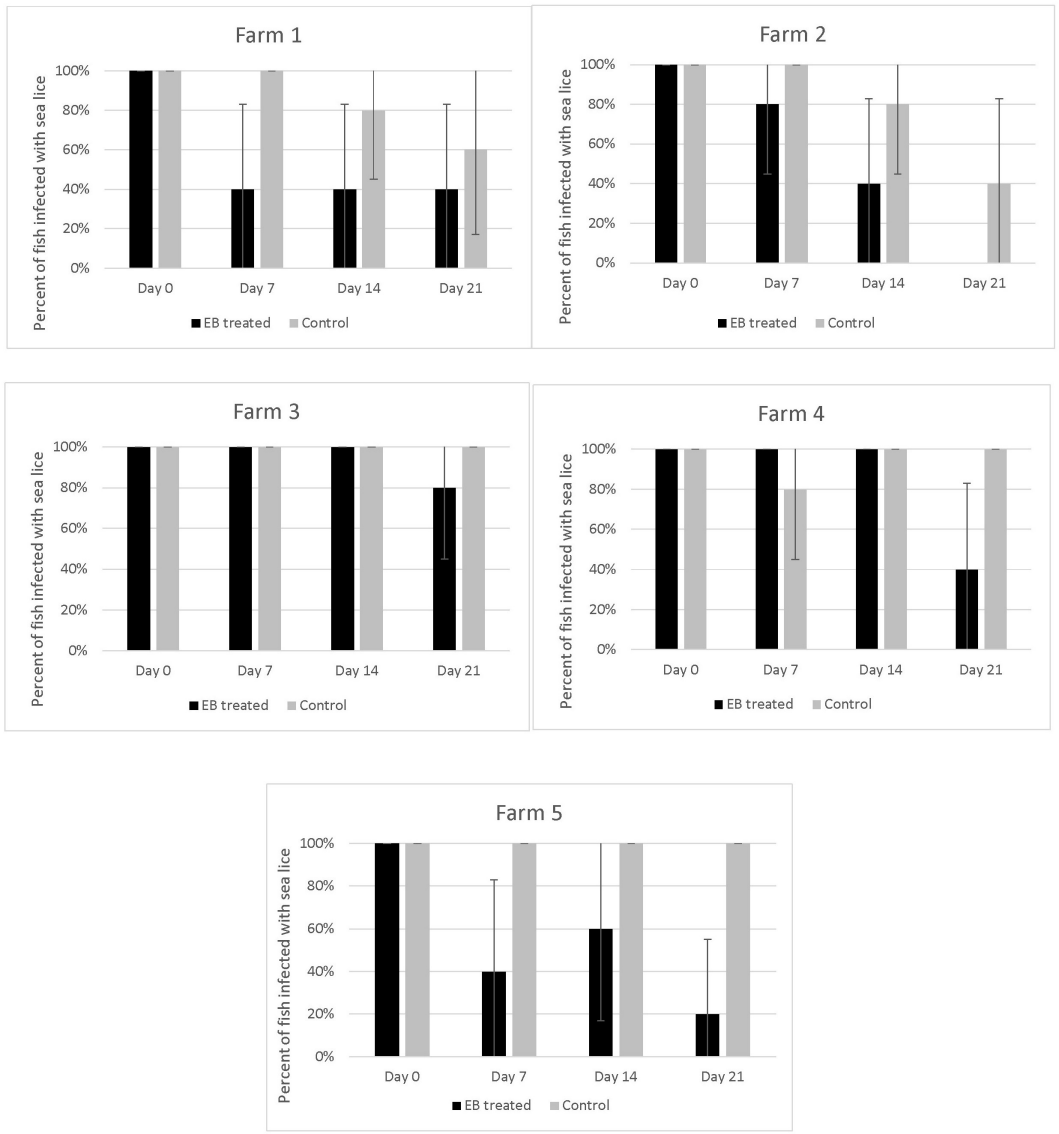
Percentage of fish with sea lice infections on five different farms taking part in an emamectin benzoate trial. Five fish were collected from both untreated (control) and treated fish on the 1st day of treatment (Day 0), and subsequently on the last day of the treatment (Day 7), day 14 and 21 of the study. A fish was defined as infected if it had any evidence of sea lice in its oral cavity (category 1 or 2 on our photographic scale). Bars indicate 95% confidence intervals (+/- 1.96* $\sqrt{\frac{p*q}{n}}\;$).

The proportion of EB treated fish with parasites declined over time on all 5 farms (Figure 4). All fish sampled on farms had some level of infection with sea lice prior to starting the treatment. Twenty–one days into the trial the proportion of infected fish on farms that had received SLICE®, with the exception of the fish on farm 3, was below 40%. Two farms (farm 1 and 2) also had a decline in the proportion of infected fish not treated with EB (Figure 4). There were some treated groups that had better overall responses to the medication relative to their untreated counterparts (i.e., farms 1, 2, and 5; Figure 4). Overall, the proportion of fish infected with sea lice (category 1 and 2) was statistically lower in the treated groups of fish compared to the non-treated groups of fish, controlling for farm effect, on all days sampled (Table 1); however, the most significant difference was observed on the last day of sampling (day 21) (Table 1).

**Table 1:** Summary of mixed effect logistic regression models for day 7, 14, and 21 of our Emamectin benzoate trials.

**Term**	**Coefficient**	**SE**	***P*-value**
**DAY 7 MIXED EFFECT LOGISTIC REGRESSION MODEL**			
**Fixed effect**			
Treatment	−2.286	1.137	0.044
**Random effect**			
Farm	0.216	0.785	0.3715
**DAY 14 MIXED EFFECT LOGISTIC REGRESSION MODEL**			
**Fixed effect**			
Treatment	−2.016	0.955	0.035
**Random effect**			
Farm	1.970	2.709	0.044
**DAY 21 MIXED EFFECT LOGISTIC REGRESSION MODEL**			
**Fixed effect**			
Treatment	−2.538	0.845	0.003
**Random effect**			
Farm	1.540	1.613	0.016

*Farms were included as a random effect in the models. The comparison of the treatment was between control and treatment groups. Five fish were included in all samples with the exception of one sampling where two fish photographs were missing*.

**Figure 5:**
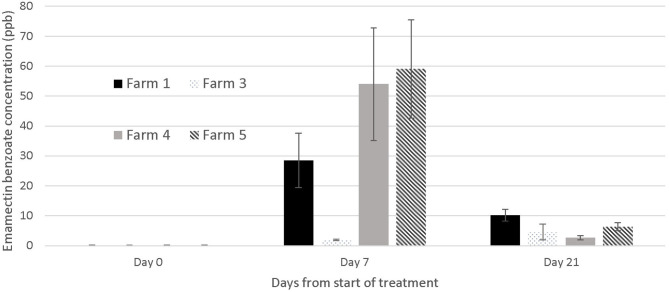
Emamectin Benzoate concentration in muscle /skin samples collected from treated fish (*n* = 5) at different days during emamectin trials on four different farms. Emamectin is reported in parts per billion (ppb) and day 0 was the 1st day of treatment (prior to the administration of the first medicated feed). Bars indicate standard error for the mean EB tissue concentration.

The concentration of EB in tissues was below the detectable limit of the LC-MS-MS analysis [2 parts per billion (ppb)] at the start of the trials, and in all fish not treated with SLICE® throughout the duration of the study, with the exception of one farm (#1) where the average EB concentration in the control fish on day 7 was 3.54 ppb (SE mean 0.578). There was a wide range of tissue concentrations on the last day of the treatment (day 7: <2 to 110.4 ppb). The range appeared to cluster by farms, with one farm appearing not to have any of its sampled fish with levels of EB above 2 parts per billion (mg/kg) (Farm #3) (Figure 5). All EB tissue concentrations were below 11 ppb in samples collected 21 days after the start of the first treatment or 14 days post treatment (Figure 5), and below 4 ppb (average 3.8, SE mean = 1.2) 21 days after the last treatment (i.e., 28 days from the start of the trials).

## Discussion

Our study identified an alternative treatment to bath chemotherapeutants for sea lice in hybrid grouper in Hong Kong. On the five farms in our study, the typical sea lice infections in hybrid grouper were reduced over time with the application of 7 days of SLICE®, a premixed product containing EB (12), at the recommended dose for salmonids (50 μg/kg/per day). The response to EB may take some time to occur as seen on farms 2 and 4 (Figure 4). Despite the fact that the level of EB in tissues of treated fish declined rapidly once the treatment ended (Figure 5) the effect of EB on the severity of infection continued to improve. This delayed response has been described in salmonids with *L. salmonis* and *C. rogercresseyi* (12), and is attributed to the mechanism of action of EB on the parasites' nervous system (12).

The response to treatment observed in this study varied by farm and was not as pronounced as what has been described in the early use of this product in the salmon industry (12, 17). Further, we observed sea lice in the oral cavity of several fish post-treatment (Figure 4), so we are confident that the EB treatment used did not completely eliminate the sea lice in all the treated fish.

Our inability to treat sea lice on grouper with 100% effectiveness (i.e., a small proportion of fish on all farms except one still had active sea lice infections after treatment) is likely due to several factors. First, the concentration of EB in fish was less than expected, given the dose we used. In fact, on one farm (Farm #3), the fish we sampled had negligible EB concentrations in their tissues on the last day of the treatment (Figure 5), suggesting a failed treatment. In salmonids, the same dose used in this study would result in a large proportion of fish with EB tissue concentrations above 60 parts per billion on the final day of the treatment (day 7) (18). The difference in the concentrations of EB in the fish from this study and salmonids may be multifactorial, and potentially played a role in the efficacy of the treatment. First, grouper and salmonids are different species cultured in very different water temperatures. The latter would have an impact on the metabolism of EB. Further, sea lice infections in grouper from Hong Kong occur in the oral cavity, which may impact the amount of medication consumed by the fish. Our records suggest a large proportion of the fish on farm 3 had moderate to severe infections prior to the treatment (50% of the fish sampled on day 0 and 7 had category 2 infections on our photographic scale; data not shown), which may explain why there was virtually no EB in the tissues of all the fish sampled on farm 3. It is also possible that the concentration of medication was not evenly distributed in the feed and this resulted in a wide variation of doses to the fish. The EB LC-MS-MS data showed a wide range of tissue concentrations at the end of the treatment both within and between farms (Figure 5) with overall EMB concentrations ranging between not detectable to 110 μg/kg), suggesting that the medication may have been consumed unevenly by the fish or dispersed on the feed unevenly resulting in the same uneven dosing effect. The actual concentration of drug administered to the fish (50 μg/kg) relative to the amount of feed was very small, so it was challenging to obtain a homogeneous distribution on the feed. We tried to standardize the protocol by mixing the feed for the farmers, but the size and the oil content of the pellets used on different farms varied and may have impacted the distribution of the medication on the feed during the hand mixing process. In the salmonid industry, all EB treatments would be incorporated into the feed at the feed mills, so the concentration of the medication delivered to the fish would be more consistent.

It should be noted that our sampling of fish may have biased our estimates of EB concentration in fish. We had to hand net fish for our samples, so we may have collected fish that were not feeding and easier to catch at the surface, rather than the healthy population of fish that typically swim deeper in the water column and are more difficult to dip net. We did throw feed in the net-pens to try to reduce this bias, but we noted that the fish swimming rapidly in the net-pen were difficult to catch. Because we potentially sampled fish that did not feed as much as others, we may have underestimated the EB concentration in the population. This underestimation could explain some of the variation on EB concentration in tissue. Further, we only sampled five fish at each time point due to financial constraints. As participants in our study were small farms and infected fish were always over 1 kg with the exception of farm 1, it was cost prohibitive to sample more fish at each time point. This fact limited the accuracy of our population estimates.

In addition to influencing our estimate of EB in fish, our sampling strategy may also have biased our estimate of sea lice on fish. This bias would have occurred in both the control and treated fish groups, and it may have biased our results toward the null (under estimation of the effect). Despite the potential sampling bias, which is inherent in field trials on small farms, all farmers in our trial agreed (personal communication) that the treatment improved grouper feeding response and were pleased with the product, despite the persistence of the mild sea lice infections in the oral cavity of some fish (Figure 5). In fact, we only had mild infection (Figure 1) in fish 1-week post treatment (day 14) except on farm 3, which still had severe infections after the EB treatment. Farm 3 was also the farm where fish had limited EB in their tissues even on the last day of treatment.

Another explanation for the lower response rate to EB observed in this study, relative to the initial reports of the effect of EB on salmon lice, is that the lice are resistant to the product. Although we were not able to measure EB resistance in our study, this hypothesis is unlikely, given that to the best of our knowledge EB has not been used by the Hong Kong fish farming community. If farmers begin to use this product and are unable to properly mix the medicated feed to deliver an accurate therapeutic dose to their fish, they may observe resistance within a few years, as has been observed in many salmon farming areas around the world (7, 8, 15, 19). Having access to a local feed mill would help reduce the risk of uneven distribution of mediation within the feed and likely improve treatment delivery.

On two of the five participating farms we observed a natural decline in sea lice infections in the control fish (Figure 4). This may have been due to environmental factors such as water temperature and salinity, which are known to impact sea lice survival (20, 21). The salinity measures on farms were all above 30%0, with a few exceptions on two farms (Figure 2). On Farm 3 we observed a salinity of 21%0 but it is unlikely that this led to a natural reduction in sea lice levels as this was the farm with some of the most severe infections. Both farms (#1 and #2), which had a natural decline in sea lice infections over time, had high salinity. Although there may have been some medication drift on farm #1, as the LC-MS-MS data suggested minimal levels of EB in the control fish on day 7, this level was very low and unlikely to be therapeutic. More likely the reduction in sea lice in the control group on these farms was associated with a decline in water temperature. These two farms reported the lowest water temperatures of the study sites (Figure 2) and the decline in sea lice paralleled the decline in water temperature. The reproductive rate of sea lice in salmon industries has been shown to be temperature dependent (20–22). It is possible that water temperature had an influence on the reproduction of the sea lice in this study. Despite the potential natural decline in sea lice on farms 1 and 2, the effect of EB was still apparent in the treatment groups (Figure 4).

One of the limitations of this study was that unlike sea lice species found on salmon, which are easy to enumerate, the species in Hong Kong, are smaller (adults are estimated to be between 2 and 4 mm in length Figure 2) and cluster with various life stages inside the mouth of the fish, therefore they are more difficult to count (Figure 1). To address this issue, we used a three-point qualitative scale for categorizing the severity of infections and most infected fish in this study had mild infections. This qualitative scale limited our ability to assess the precise reduction in parasite numbers post-treatment. Despite this limitation in counting individual sea lice in the mouth of the fish, we were still able to measure changes in sea lice clusters and lesions.

Within 2 weeks of the last day of treatment, the level of EB in tissues was below 10 ppb. This decline in drug concentrations was steeper than what is reported in salmonids (11, 23–25), and was likely due to the elevated water temperature in our study (range between 19.7 and 29.1°C). By day 28 of our experiment [21 days after the last treatment the average levels of EB were 3.8 ppb (SE = 1.14)], which suggests the withdrawal period of 21 days recommended for SLICE® at a dose of 50 μg EB/kg of fish per day for 7 days (12) could be used for grouper. The levels of EB in our fish tissue samples on day 21 were well below the 42 ppb maximum residue limit used in some countries (26).

In conclusion, EB appears to be effective for reducing sea lice in the oral cavity of hybrid grouper in Hong Kong. EB has potential to be used within the Asian grouper aquaculture industry with good results if protocols of administration are monitored closely. Response to treatment may be improved by refining the delivery of the product to fish and treating animals early in the infection process to ensure fish are still eating adequately to receive the proper dose of medication. Although we observed a decline in sea lice infestation on most farms after the use of SLICE®, a small percentage of treated fish still had parasites on all but one farm. The specific dose required for complete elimination of sea lice needs further investigation. This is important because exposing parasites to subtherapeutic levels of EB could exacerbate the risk that resistance develops to this therapeutant (27). Lastly, the environmental impact of using SLICE on farms in Asia should be assessed as it has recently been demonstrated to reduce the abundance of crustaceans in the benthic environment around salmon farms in Scotland (28).

## Data Availability Statement

The raw data supporting the conclusions of this article will be made available by the authors, without undue reservation.

## Ethics Statement

The animal study was reviewed and approved by City University of Hong Kong Ethics Committee. Written informed consent was obtained from the owners for the participation of their animals in this study.

## Author Contributions

SS-H was the project leader and wrote the first draft of the manuscript. TC, SC, KC, CL, and KL were the technical team who conducted the trails on the farms. WF was the graduate student who assisted with the writing of the manuscript and the data analysis. GBG assisted with the parasite identification and quantification. GBG contribute with article editing. All authors contributed to the article and approved the submitted version.

## Conflict of Interest

The authors declare that the research was conducted in the absence of any commercial or financial relationships that could be construed as a potential conflict of interest.

## Footnotes

^1^One farm (farm 2) did not want us to conduct lethal sampling, so we did not submit samples for LC-MS-MS evaluation.
